# Uptake and Competition Among Biosimilar Biological Products in the US Medicare Fee-for-Service Population

**DOI:** 10.1007/s11606-022-07670-7

**Published:** 2022-06-01

**Authors:** Steven Kozlowski, Natasha Flowers, Andrew Kwist, Sarah K. Dutcher, Michael Wernecke, Jeffrey A. Kelman, David J. Graham

**Affiliations:** 1 Center for Drug Evaluation and Research, US Food and Drug Administration, Silver Spring, MD USA; 2 Acumen, LLC, Burlingame, CA USA; 3 Centers for Medicare & Medicaid Services, Washington, DC, USA

## INTRODUCTION

The Biologics Price Competition and Innovation Act of 2009 created an abbreviated US approval pathway for biosimilar and interchangeable biological products. Biological products are usually large, complex molecules, in contrast to typical simpler, small-molecule drugs. A biosimilar is a biological product that is highly similar to and has no clinically meaningful differences from a reference product. As of January, 1, 2022, the FDA had approved 33 biosimilars, with at least 21 currently marketed in the USA.^1^ For generic drugs, which can receive a period of market exclusivity, the order of market entry impacts market share, with the first product usually having higher uptake than subsequent products.^2,3^ There is no comparable market exclusivity for non-interchangeable biosimilars. Our study evaluated factors that may influence biosimilar uptake, including market order entry, clinical setting, and initial publicized pricing discounts.

## METHODS

We evaluated uptake of 13 biosimilar products from June 3, 2015, to July 1, 2021, in the Medicare Fee-for-Service population. We included all first- and second-marketed biosimilars with at least 12 months of available claims data plus three additional months for reporting lag. No interchangeable products met this requirement. Market share is calculated as the number of biosimilar administrations out of the total administrations of the product, defined as the reference product and any marketed biosimilars (including tbo-filgrastim, approved under FDA’s full Biologics License Application before establishment of the biosimilar approval pathway^1^). We present biosimilar uptake as both an ongoing monthly metric and a cumulative metric for the initial 12 months of marketing of each product. The start of marketing was defined as the first month of product administration within Medicare, as identified via HCPCS codes and modifiers on Medicare Part B claims. We also evaluated the care setting of biosimilar administration and initial publicized pricing discounts relative to reference product^1^.

## RESULTS

**Figure 1.**
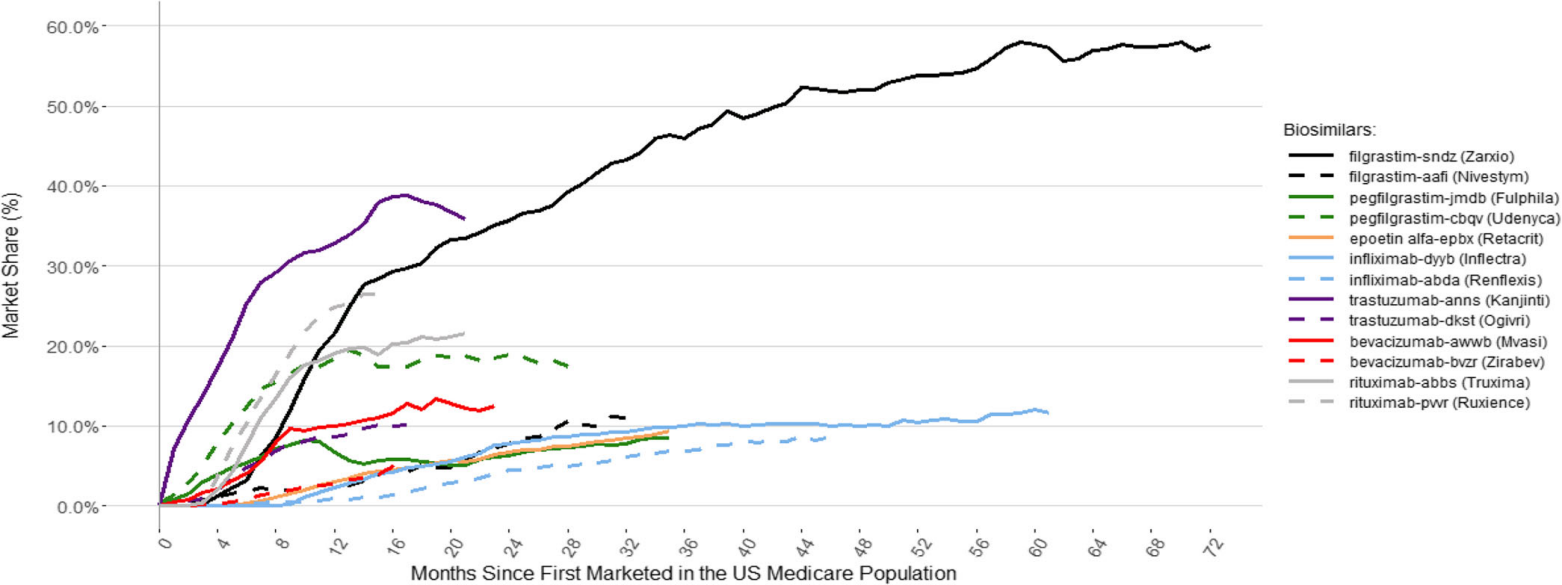
Monthly percent of administrations for each biosimilar within a product class. The number of administrations for each biosimilar identified in the legend is shown as a percentage of the total number of administrations of that product class on a monthly basis. The total administration by product class includes the reference product, all biosimilars, and related biological products, if applicable (i.e., tbo-filgrastim)

**Figure 2.**
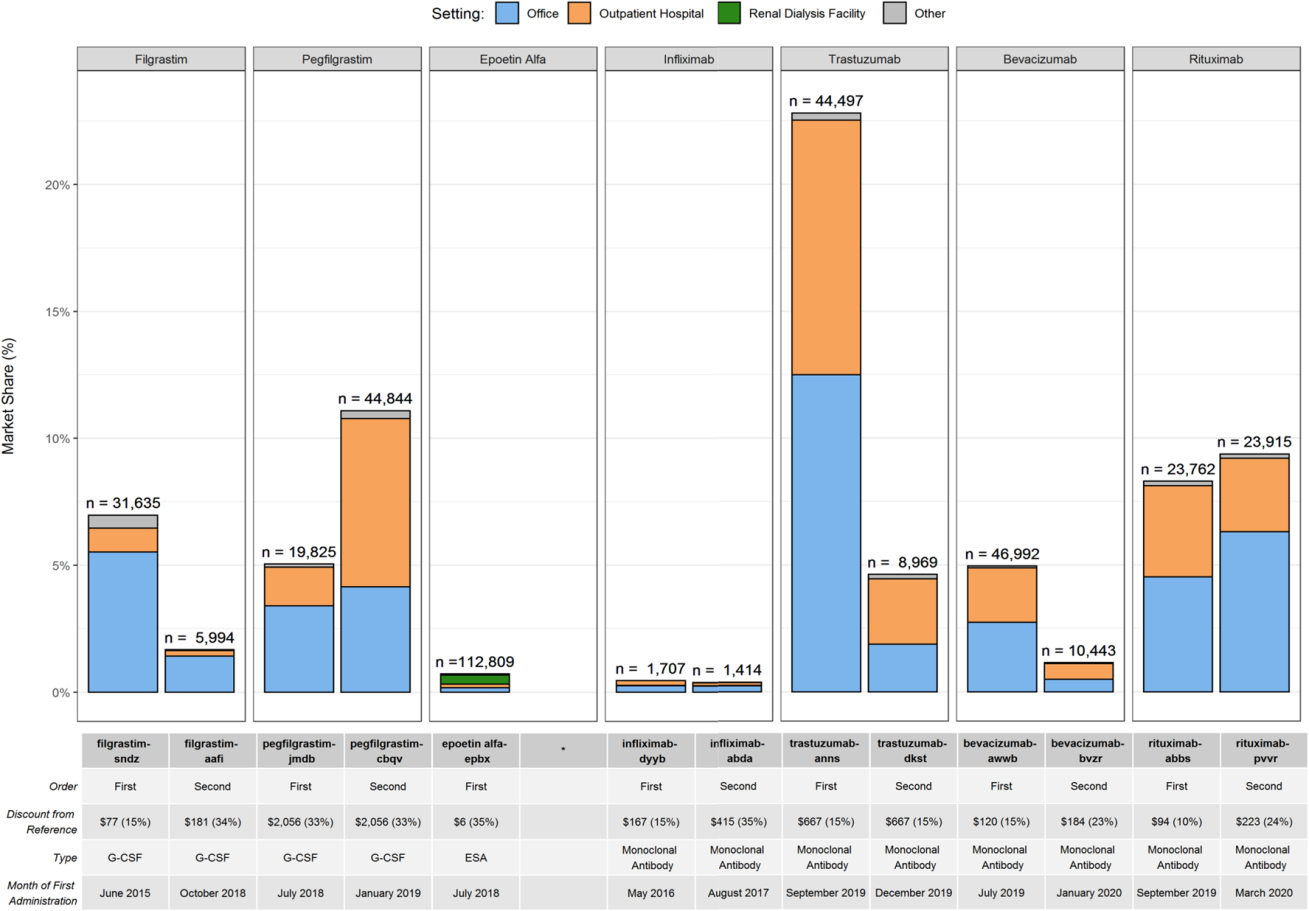
Cumulative uptake percentage of biosimilars over 12 months since first product administration, by care setting**.** The percentage of total administrations by product class is shown for each biosimilar product over its first 12 months of marketing. Biosimilar products are identified by 1st and 2nd market entries and match the products in those categories in Figure 1. The percent of total administrations is broken up by the setting of the administration as indicated; the ‘Other’ category includes settings such as ambulatory surgical center, home, and nursing facility, among others. The month of first observed biosimilar administration, percent discount from reference product in wholesale acquisition cost, total administrations over 12 months, and product type are shown for each product . ESA, erythropoiesis-stimulating agents; G-CSF, granulocyte colony-stimulating factor. *No second biosimilar with complete 12 months of data.

Biosimilar uptake varied by product, entry order, and care setting. Of the products marketed longest, filgrastim-sndz accounted for over 50% of monthly filgrastim administrations, whereas infliximab-dyyb accounted for approximately 10% of monthly infliximab administrations (Figure 1). Infliximab and epoetin alfa biosimilars both had low cumulative market shares after 12 months of marketing (<1%, Figure 2). The first filgrastim, trastuzumab, and bevacizumab biosimilars had higher uptake than the second, whereas the second pegfilgrastim and rituximab biosimilars had higher uptake than the first. The initial pricing discount for the second pegfilgrastim biosimilar did not differ from the first, while the discount of the second rituximab was higher than the first^1^ (Figure 2). Care setting also differed by entry order: the second pegfilgrastim, trastuzumab, and bevacizumab biosimilars had relatively more outpatient hospital administrations than their respective first biosimilars, whereas second filgrastim and rituximab biosimilars had relatively fewer outpatient hospital administrations than their respective first biosimilars.

## DISCUSSION

We found that biosimilar uptake was generally lower than that reported for generic drugs. Generics that entered the market in 2013–2014 took 88% of the market within 1 year on average, compared with approximately 1 to 33% for the studied biosimilars.^4^ While early-marketed generic drugs often perform better than later ones,^2,3^ and early market entry of new molecular entities has been shown to be beneficial when controlling for other factors,^5^ the impact of order of entry was mixed for the studied biosimilars. Although first biosimilars had higher market uptake than second biosimilars for several reference products, the second pegfilgrastim and rituximab biosimilars performed better than their respective first biosimilars. We observed no clear patterns that would explain this across both products; the second pegfilgrastim biosimilar had a notably larger share of outpatient hospital administrations than its first biosimilar, while the second rituximab biosimilar did not. The initial publicized pricing discounts also did not correlate with uptake. Of note, publicized discounts may not reflect actual product pricing, which could be a factor in uptake.^6^ Another study limitation is that findings from Medicare Fee-for-Service data are not necessarily generalizable to commercial payers in Medicare Advantage. Our study suggests non-interchangeable biosimilars behave differently than generics. We did not observe consistent trends in biosimilar uptake by order of entry, care setting, or pricing. More study is needed to understand and leverage the factors for biosimilar uptake that can lower costs and increase access to lifesaving biological products.
